# Epigenetic silencing of XAF1 in high-grade gliomas is associated with IDH1 status and improved clinical outcome

**DOI:** 10.18632/oncotarget.14748

**Published:** 2017-01-19

**Authors:** Thomas R. Reich, Olivier J. Switzeny, Mirjam Renovanz, Clemens Sommer, Bernd Kaina, Markus Christmann, Maja T. Tomicic

**Affiliations:** ^1^ Department of Toxicology, University Medical Center, D-55131 Mainz, Germany; ^2^ Department of Neurosurgery, University Medical Center, D-55131 Mainz, Germany; ^3^ Department of Neuropathology, University Medical Center, D-55131 Mainz, Germany

**Keywords:** high-grade glioma, glioblastoma, temozolomide, XAF1 promoter methylation, IDH1

## Abstract

XAF1 (X-linked inhibitor of apoptosis (XIAP)-associated factor 1) is a tumor suppressor that counteracts the anti-apoptotic effects of XIAP and can sensitize cells to cell death triggering events. XAF1 knockdown abrogated the temozolomide (TMZ)-induced G2-arrest and prevented TMZ-induced apoptosis in the glioblastoma (GB) cell line LN229. Promoter methylation of *XAF1* was found to be inversely correlated with mRNA expression in GB cells. We analyzed *XAF1* methylation in a panel of 16 GB cell lines and 80 patients with first-diagnosed WHO grade III/IV high-grade gliomas using methylation-sensitive high-resolution melt (MS-HRM) analysis. In those patients, *XAF1* promoter methylation was strongly associated with enhanced progression free and overall survival. Interestingly, *XAF1* promoter methylation was strictly correlated with the occurrence of *IDH1* mutations, indicating a causal link to the *IDH1* mutant phenotype. *XAF1* methylation was observed in 18 grade III tumors all of which showed heterozygous mutations in the *IDH1* gene. 17 harbored a mutation leading to an arginine > histidine (R132H) and one carried a mutation causing an arginine > glycine (R132G) substitution. Furthermore, six out of six recurrent and *IDH1* mutated grade III tumors also showed *XAF1* promoter methylation. The data demonstrate that *XAF1* promoter methylation determined by MS-HRM is a robust and precise indicator of *IDH1* mutations in grade III gliomas. It is useful for complementing the immunohistochemistry-based detection of mutant IDH, uncovering rare 2-HG-producing *IDH1* and potentially *IDH2* mutations. The MS-HRM-based detection of *XAF1* methylation could therefore be a reliable tool in assisting the sub-classification of high-grade gliomas.

## INTRODUCTION

In accordance with the 2007 World Health Organization (WHO) guidelines [1], tumors of the central nervous system have been classified by histological criteria. This not only defines the tumor type, but also the grade of malignancy. High-grade gliomas (HGG) or malignant gliomas mainly consist of WHO grade III and grade IV tumors. Glioblastomas (GB) (WHO grade IV) account for 60–70% of HGG. Among WHO grade III tumors anaplastic astrocytomas (AA) account for 10–15% and anaplastic oligodendrogliomas (AO) together with anaplastic oligoastrocytomas (AOA) for 10% of HGG [2]. Due to massive progress in the knowledge of the genetic basis of tumorigenesis, a major revision of the HGG classification was necessary as suggested by the Haarlem consensus guidelines for nervous system tumor classification and grading [3], which finally culminated in the 2016 update of the WHO guidelines [4]. In the new classification, histology and molecular parameters are used to define the different tumor entities. The 2016 WHO classification is mainly based on three molecular markers: isocitrate dehydrogenase 1/2 (*IDH1*/*IDH2*) mutations, allelic loss of chromosome 1p and 19q, as well as somatic mutations in the alpha thalassemia/mental retardation syndrome X-linked (*ATRX*) gene.

Of utmost importance for molecular classification and prognosis in gliomas is the status of the *IDH1* gene. Specific mutations in this gene are associated with a strongly improved clinical outcome [5, 6]. *IDH1* mutations are found in more than 70% of WHO grade II/ III astrocytomas and oligodendrogliomas as well as in secondary GB [6], therefore linking *IDH1* mutation predominantly to lower grade and grade III gliomas, as well as GB having evolved from the aforementioned. The most common mutation found in *IDH1* leads to an arginine to histidine substitution (R132H) in the active site of the protein [7]. As gain-of-function mutation, this enables the enzyme to produce 2–hydroxyglutarate (2-HG) instead of its normal product α-ketoglutarate (αKG) [8]. This oncometabolite is sufficient to establish the glioma CpG island methylator phenotype (G-CIMP) [9] that is associated with distinct molecular subgroups of gliomas, linked to younger age at diagnosis and better prognosis [10]. The CIMP is characterized by an extensive, coordinated hypermethylation at specific gene loci. Also, additional mutations in the *IDH2* gene, apart from *IDH2* R172 (e.g. R140), which in gliomas occur less frequently than those in the *IDH1* gene, give the same phenotype [6, 11–13].

The current treatment of HGG consists of a maximum safe resection followed by radiotherapy with concomitant or adjuvant temozolomide (TMZ) administration [14]. TMZ exerts its cytotoxic effect by the induction of O^6^-methylguanine, which, in the presence of the mismatch repair, ultimately leads to the formation of DNA double-strand breaks (DSB) and cell death [15]. O^6^-methylguanine can be repaired by the DNA repair enzyme O^6^-methylguanine-DNA methyltransferase (MGMT). Since MGMT expression is inhibited by methylation of its promoter, and *MGMT* promoter methylation correlates with enhanced overall survival (OS) and progression free survival (PFS), the methylation status of *MGMT* is used as a predictive marker for glioma therapy [16]. Previously, we observed that members of the inhibitor of apoptosis (IAP) family, Survivin and XIAP, can also protect malignant glioma cells from anticancer therapy [17]. An important factor, blocking the anti-apoptotic effect of Survivin and XIAP by targeting the proteins for proteasomal degradation, is the tumor suppressor X-linked inhibitor of apoptosis (XIAP)-associated factor 1 (XAF1) [18, 19]. XAF1 is ubiquitously expressed in normal tissue, while in cancer cells its expression is often reduced [20]. XAF1 expression is absent or reduced in gastric [21], colon [21], ovarian [22], pancreatic [23], esophageal [24], hepatic [25], melanoma [26], and urogenital tumors [27–29], and is largely regulated by promoter CpG dinucleotide hypermethylation, which leads to gene silencing [21, 24, 29]. XAF1 protein expression was shown to suppress tumor cell growth and enhance cellular response to various apoptotic stimuli, such as 5-fluorouracil, etoposide, H_2_O_2_, γ-irradiation, UV light and TNFα, whereas knockdown of its expression protected cells from the stressors [30]. Furthermore, enhanced XAF1 expression inhibited cell proliferation and induced apoptosis in HCC cells [31] and in gastric and colon cancer xenografts [32, 33]. In gastric cells, this seems to be associated with a role of XAF1 in inducing G2/M arrest [33]. This cell cycle arrest is explained by a direct interaction with the activated checkpoint kinase 1 (CHK1), leading to inactivation of Cdc25C in the Cdc2-cyclin B complex [34]. Although there are no reports describing a direct impact of *XAF1* methylation on radiation sensitivity, there are few hints indicating that an increased XAF1 expression sensitizes cells towards ionizing radiation (IR). An association was found between a high expression of XAF1 and induction of apoptosis based on an ubiquitin-dependent degradation of CHK1 by the XAF1-XIAP complex, leading to enhanced radiation sensitivity [35].

The main goal of the present study was to elucidate whether *XAF1* is epigenetically silenced in HGG and whether the methylation status of the *XAF1* promoter can serve as a prognostic and/or predictive marker. Therefore, we utilized methylation-sensitive high-resolution melt (MS-HRM) analysis [36] that was recently shown to provide excellent prognostic outcomes in *MGMT* promoter methylation studies [37]. By adopting this method, we analyzed the CpG methylation in a distinct promoter region of *XAF1* in HGG cell lines and in 80 formalin-fixed, paraffin-embedded (FFPE) tumor samples. These samples were obtained from HGG patients, prior to the standard IR/TMZ therapy. Additionally, 16 samples of recurrent HGG were analyzed.

## RESULTS

### Influence of XAF1 knockdown on the response to TMZ

**Figure 1:**
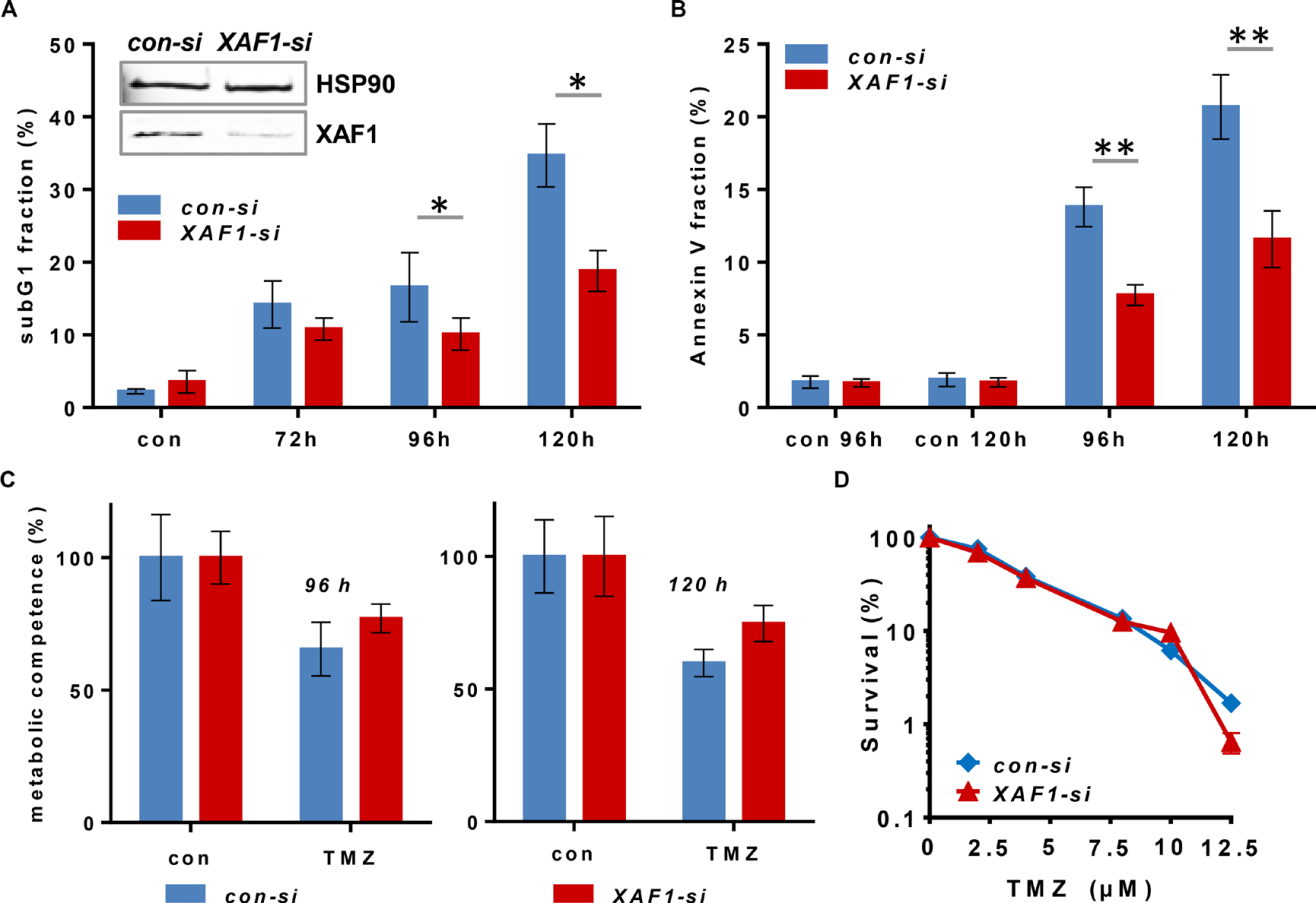
Cytotoxicity end points upon siRNA-mediated *XAF1* silencing in malignant glioma cells exposed to TMZ *XAF1* knockdown was performed 24 h prior to the treatment of the cells. Time points indicated refer to the treatment time (**A**) Flow cytometric analysis of apoptosis induction (subG1) is shown in LN229 cells after *XAF1* knockdown (*XAF1-si*) and transfection with non-coding siRNA (*con-si*). Cells were treated with 100 μM TMZ and fixed after the time points indicated. After PI staining, the subG1 fraction was determined. Error bars indicate the SD in three independent experiments in duplicates (*N* = 3). Data were analyzed for statistically significant differences by two-tailed *t-test*, comparing target *XAF1-si* *vs*. *con-si*. A *p-value* of < 0.05 was considered to be statistically significant (*). The efficiency of siRNA-mediated *XAF1* knockdown was verified by western blot analysis 24 h after transfection. HSP90 was used as loading control. (**B**) Flow cytometric analysis of annexin V-FITC/PI - stained LN229 cells upon transfection with *con-si* and *XAF1-si* RNA and exposure to 100 μM TMZ for 96 and 120 h. The corresponding unexposed controls (con 96 h, con 120 h) are shown. Error bars indicate the SD in two independent experiments in duplicates (*N* = 2). Data were analyzed for statistically significant differences by two-tailed *t-test*, comparing target *XAF1-si* *vs*. *con-si*. A *p-value* of < 0.01 was considered to be statistically significant (**). (**C**) The cell viability (metabolic competence) upon treatment with 100 μM TMZ was determined by MTT assay in *con-si* and *XAF1-si* transfected LN229 cells. Technical triplicates at 96 and 120 h are shown. (**D**) Colony forming assay of LN229 cells transfected with *con-si* and *XAF1-si* RNA, exposed to increasing TMZ concentrations (semi-logarithmic scale). Two experiments in duplicates are shown. The unexposed controls were set to 100%.

**Figure 2:**
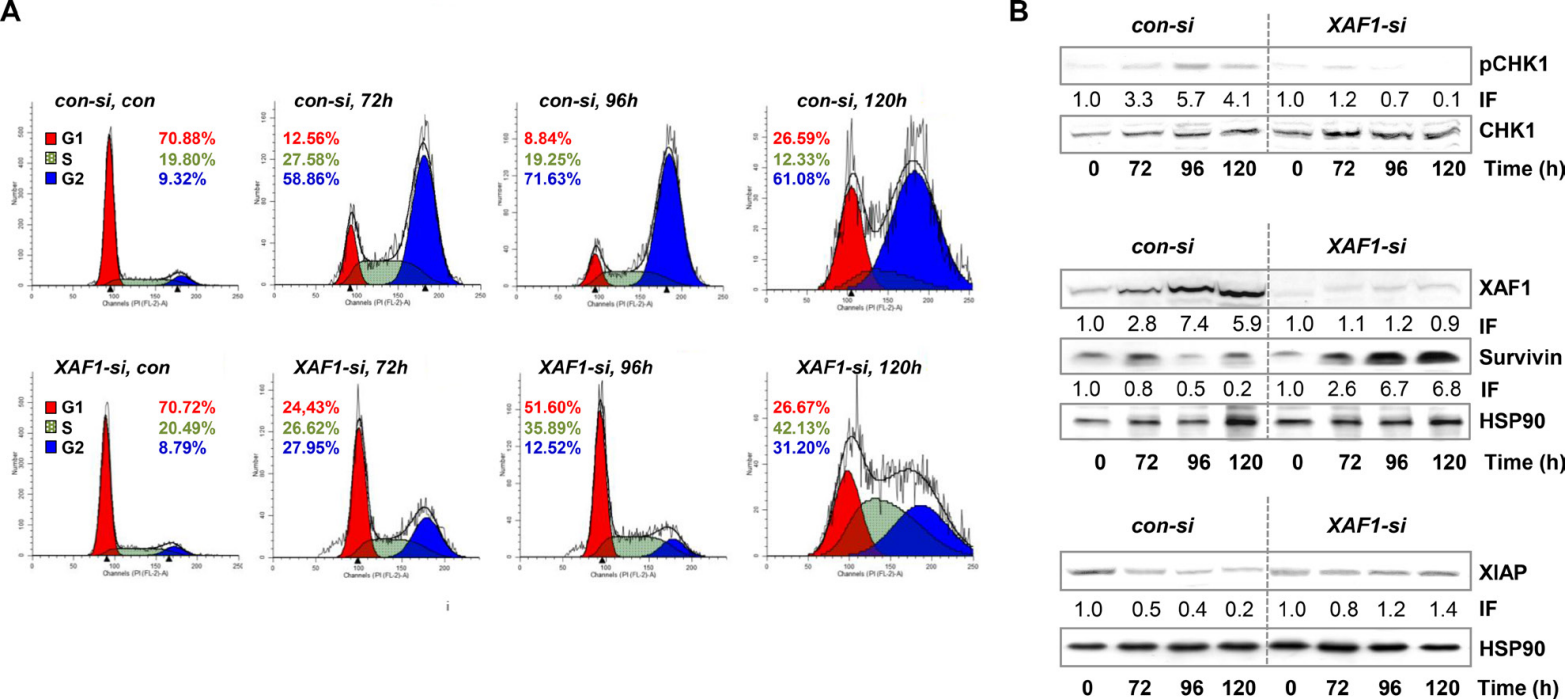
Cell cycle distribution and protein expression upon *XAF1* knockdown (**A**) The cell cycle distribution upon *XAF1* knockdown and transfection with *con-si*, was analyzed in a time frame of 72–120 h after exposure to 100 μM TMZ. Automated cell cycle analysis was performed using ModFit LT 3.3. The representative histograms of one out of three experiments are shown. (**B**) Protein levels of XAF1, XIAP, Survivin and phosphorylated CHK1 (pCHK1) with HSP90 as loading control, were determined upon TMZ treatment (100 μM) and compared in *XAF1-si* and *con-si* transfected LN229 cells by western blot analysis. One representative blot out of three independent experiments is shown. The corresponding protein expression was quantified by densitometric analysis in relation to the loading control. IF = induction factor.

To analyze a putative impact of XAF1 on TMZ-induced cell death and/or cell cycle progression, XAF1 expression was silenced by siRNA in the GB cell line LN229. The effect of XAF1 knockdown on induction of apoptotic cell death (Figure 1) and cell cycle distribution (Figure 2) upon exposure to TMZ was determined. Measurement of the SubG1 fraction indicated that XAF1 knockdown protects *in vitro* against TMZ-induced apoptosis (Figure 1A). The data were confirmed by annexin V/PI double staining (Figure 1B), showing specific reduction of TMZ-induced apoptosis in *XAF1-si* transfected LN229 cells. Necrosis was only marginally induced (< 5%; data not shown). Furthermore, *XAF1-si* transfected LN229 cells had a higher metabolic competence upon exposure to TMZ than the *con-si* transfected cells (Figure 1C). These *in vitro* data are in accordance with other reports, demonstrating that enhanced XAF1 expression induces apoptosis in tumor cells [31] and in xenografts [32, 33].

In comparison to short-term exposure (subG1, annexin V, MTT), there was no difference in colony formation (reproductive cell survival) between *XAF1-si* and *con-si* transfected TMZ-exposed LN229 cells (Figure 1D). This indicates that for long-term survival differences, the impact of XAF1 on cellular processes beside apoptosis, for instance on cell cycle progression, plays a predominant role. Thus, we observed that upon *XAF1* knockdown, LN229 cells exposed to TMZ did not accumulate in the G2-phase. The cells rather accumulated in the G1-phase (72–96 h) and later-on (120 h upon TMZ exposure) in the S- and G2-phase (Figure 2A).

Furthermore, the expression of XAF1, XIAP, and Survivin, as well as the activation of CHK1 (which is important for TMZ-induced G2-arrest) was analyzed upon TMZ treatment depending on *XAF1* silencing (Figure 2B). The data show that *XAF1* knockdown led to the stabilization of Survivin, as compared to the *con-si* transfected TMZ-exposed cells, and also protected from down-regulation (degradation) of XIAP. Importantly, activation (phosphorylation) of CHK1 (pCHK1) was reduced upon *XAF1* knockdown.

Since the *in vitro* data in GB cells suggest an impact of XAF1 on the sensitivity to TMZ, we addressed the question whether XAF1 expression plays a role in the course of disease of malignant gliomas.

### XAF1 promoter methylation is predictive for XAF1 expression

**Figure 3:**
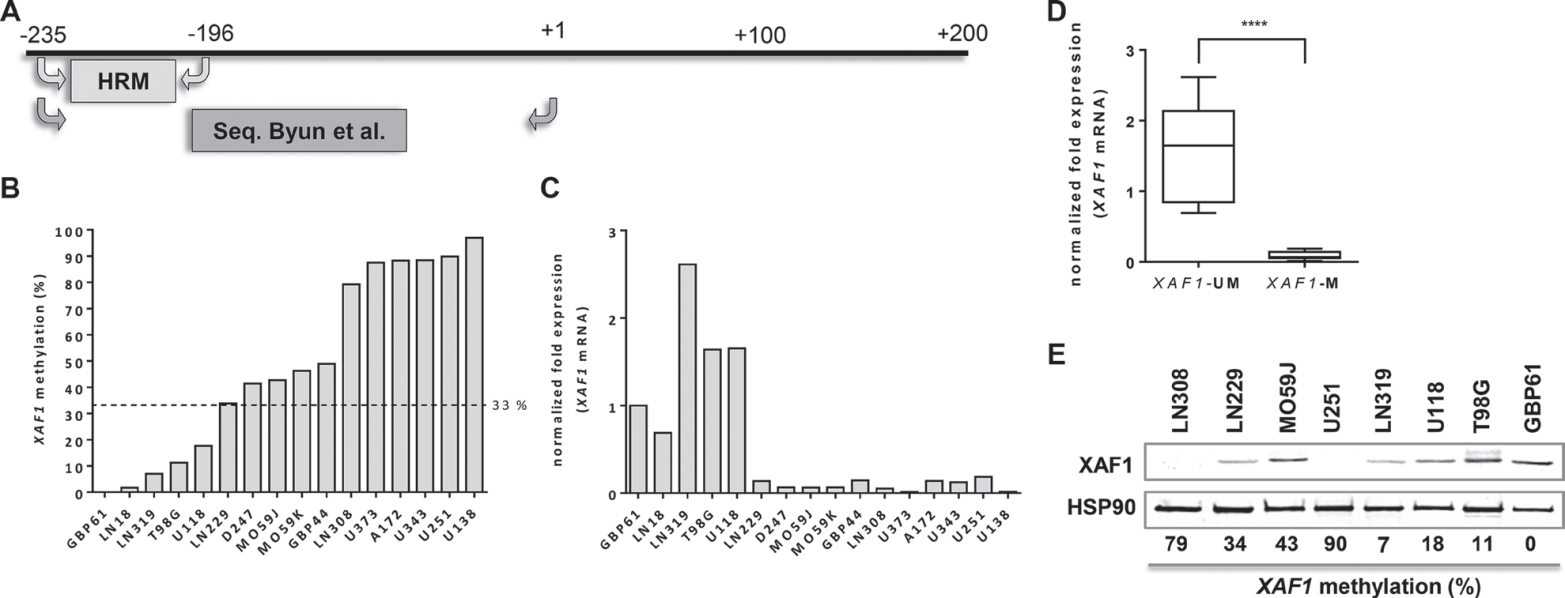
*XAF1* methylation in glioma cell lines (**A**) Schematic promoter region of *XAF1* with the location of MS-HRM primers used for this analysis and sequencing primers used by Byun *et al*. [46] (**B**) *XAF1* promoter methylation in 16 GB cell lines analyzed by MS-HRM. The cell lines were ordered by the percentage methylation of the analyzed *XAF1* promoter fragment starting with the lowest methylation value (from left to right). MS-HRM was carried out in technical duplicates of bisulfate-converted DNA of each cell line. The dotted line indicates the threshold of methylation which was applied for grouping. Samples with methylation values above 33% were considered as “methylated”. (**C**) *XAF1* mRNA expression in 16 GB cell lines as determined by quantitative RT-PCR. The expression (relative to GBP61) of *XAF1* mRNA is shown, normalized to *ACTB* and *ENOX2*. Expression in each cell line was detected in technical triplicates (**D**) The grouping of the 16 GB cell lines according to *XAF1* methylation status. Cell lines with a methylation value above 33% were considered to be methylated (M), whereas those with a methylation level of ≤ 33% were set as unmethylated (UM). Statistical significance for the difference in both groups was tested by a two-tailed *t-test* (*p-value* < 0.0001, (****)). (**E**) XAF1 protein expression in selected GB cell lines with HSP90 loading control. The *XAF1* promoter methylation percentages as determined by MS-HRM are indicated below the blot.

To analyze whether XAF1 expression represents a prognostic marker in HGGs, we established a MS-HRM assay for the detection of *XAF1* promoter methylation, using bisulfate-converted methylated and non-methylated genomic DNA. The primer pair used flanks three CpGs in the region -196 to -235 (Figure 3A), which overlaps with a region already found to be responsible for *XAF1* silencing in gastric cancer. MS-HRM was used for the methylation analysis of the selected *XAF1* promoter region in 16 HGG cell lines (Figure 3B). Six cell lines showed an overall high methylation level (79–100%). Four cell lines showed an intermediate methylation (34–78%), whereas five cell lines showed a low methylation (< 33%).

To examine the methylation status of these CpGs in more detail, the region amplified by MS-HRM was analyzed by pyrosequencing in six cell lines (Supplementary Table 1, Supplementary Figure 1). Methylation levels detected by pyrosequencing and MS-HRM showed a strong correlation (*r* = 0.965; *p* = 0.0018), verifying MS-HRM as accurate method for methylation analysis. Detailed methylation levels for all three CpGs analyzed are provided in Supplementary Table 1.

In order to identify a cut-off level, the promoter methylation of the cell lines was compared to the corresponding *XAF1* mRNA expression in 16 HGG cell lines (Figure 3C). *XAF1* mRNA was only detectable for methylation levels of ≤ 33%. LN229 cells exhibited an average methylation of 34% with almost non-detectable mRNA expression. Thereby using 33% as threshold for methylation, we were able to define cell lines as XAF1-methylated (XAF1-M) and XAF1-unmethylated (XAF1-UM). Applying this threshold to the glioma cell line panel, a significantly higher *XAF1* mRNA expression was found in the unmethylated group (Figure 3D, *p* < 0.0001).

In addition to the mRNA expression, also XAF1 protein levels were determined in selected cell lines. In line with the previous findings, cell lines with no or low promoter methylation (LN319, U118, T98G, GBP61) showed expression of the XAF1 protein. In contrast, cell lines with a highly methylated *XAF1* promoter (U251, LN308) showed no detectable expression (Figure 3E). The situation is less clear for the cell lines showing an intermediate methylation level (e.g. LN229); in this case, expression of the protein was still evident. Since we observed the best stratification between *XAF1* mRNA expressing and *XAF1* mRNA non-expressing cell lines, using a cut-off level of · 33%, we used this value for the analysis of the impact of *XAF1* methylation on clinical parameters in HGG patients.

### XAF1 promoter methylation in malignant brain tumors

**Figure 4:**
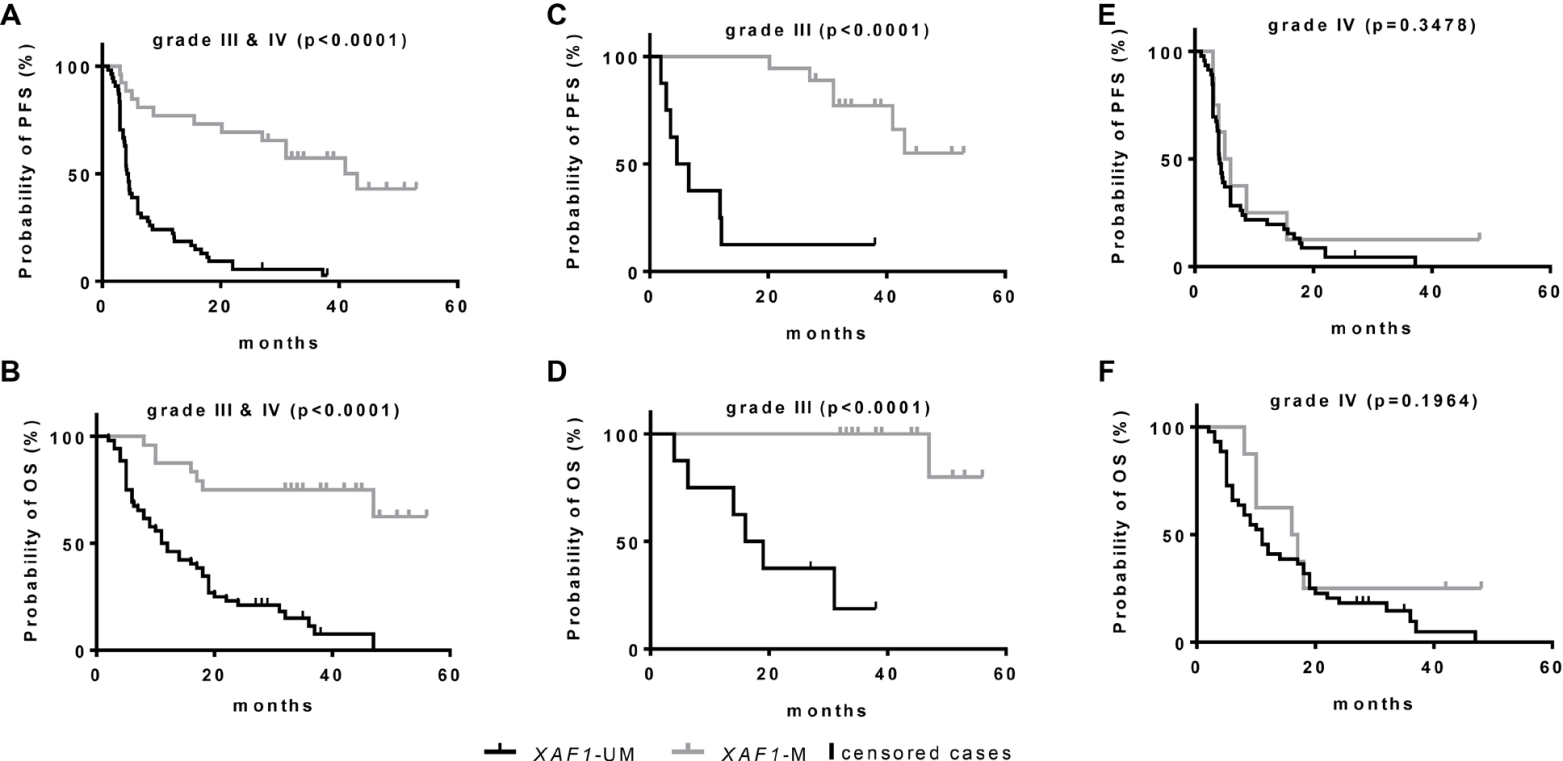
Kaplan-Meier survival estimates for HGG patients according to *XAF1* promoter methylation state Kaplan-Meier survival estimates were calculated in a group of 80 HGG patients stratified for methylated (M) and unmethylated (UM) *XAF1* promoter, determined by MS-HRM. Survival probability was calculated for all patients (grade III and grade IV) using the PFS (**A**) and OS (**B**) and for subgroups, further stratified for the histological tumor grade in grade III tumors (**C**, **D**) and in tumors of grade IV (**E**, **F**). *P*-values indicate the statistical significance of the differences in both groups (log-rank test).

Having confirmed the biological relevance of the methylation in the three CpGs analyzed, for *XAF1* mRNA and protein expression, we evaluated the *XAF1* status in 80 HGG tumor samples. A total of 26 patients (32.5%) showed a methylated *XAF1* promoter in the tumor tissue analyzed with no gender prevalence (Table 1). *XAF1* methylation occurred more often in patients with an age below 70 years at diagnosis. To test the influence of the *XAF1* methylation on the PFS and OS, promoter methylation status was used for stratification in Kaplan-Meier survival curves (Figure 4). Patients were dichotomized according to the defined threshold, as either *XAF1*-M or *XAF1*-UM. Strongly increased PFS (*p* < 0.0001; Figure 4A) and OS (*p* < 0.0001; Figure 4B) were observed for *XAF1*-M patients. While the observed median PFS of all patients with an *XAF1*-UM state was 4.4 months, the group with *XAF1* methylation positive tumors showed a significantly increased PFS of 41.0 months (see Table 2). Although Kaplan-Meier survival curves also showed a significantly increased OS for *XAF1*M, the exact median survival could not be calculated, as the survival curve did not drop below 50% at the end of the observation period. Stratifying according to the tumor histology (AA, AO and AOA vs. GB), the survival differences observed were specific for grade III tumor entities (*p* < 0.0001; Figure 4C/4D), as they did not differ significantly in grade IV tumors (GB) (PFS: *p* = 0.3478; Figure 4E, OS: *p* = 0.1964; Figure 4F). Thus, *XAF1* methylation status represents a prognostic marker for grade III gliomas, being positively linked to PFS (*r* = 0.562, *p* < 0.01) and OS (*r* = 0.525, *p* < 0.01). In line with this, the median PFS is longer in *XAF1*-M patients with grade III tumors when compared to the *XAF1*-UM group (43.0 vs. 6.6 months), whereas it is not prolonged in grade IV patients (4.1 vs. 5.0 months) (Table 2).

**Table 1:** Patient's characteristics

*Characteristics*	N	*XAF1*-M % (N)	*IDH1*mut % (N)
All patients	80	32.5 (26)	25.0 (20)
Women	24	29.2 (7)	25.0 (6)
Men	56	33.9 (19)	25.0 (14)
Age < 70	56	41.1 (23)	35.7 (20)
Age ≥ 70	24	12.5 (3)	0.0 (0)
Grade III*	26	69.2 (18)	69.2 (18)
Grade IV*	54	14.8 (8)	3.7 (2)
AA, AOA, AO *IDH1*mut	18	100.0 (18)	100.0 (18)
AA, AOA, AO *IDH1*wt	8	0.0 (0)	0.0 (0)
GB IDH1mut	2	100.0 (2)	100.0 (2)
GB IDH1wt	54	11.1 (6)	0.0 (0)

*XAF1* promoter methylation (*XAF1*-M) was determined by MS-HRM and *IDH1* mutation.

(*IDH1*mut) by pyrosequencing and IHC. *Histological tumor grade: AA: anaplastic astrocytoma;

AO: anaplastic oligodendroglioma; AOA: anaplastic oligoastrocytoma; GB: glioblastoma.

**Table 2:** Kaplan-Meier survival estimates for HGG patients

*PFS*	*all*		grade III*		grade IV*		AA, AOA, AO *IDH1*mut		AA, AOA, AO *IDH1*wt	
*XAF1*	mean	median	mean	median	mean	median	mean	median	mean	median
UM	8.7	4.4	13.3	6.6	7.8	4.1	-	-	10.2	4.6
M	32.7	41.0	42.0	43.0	11.0	5.0	44.7	n.d	-	-
p	0.000		0.000		0.348		n.d.		n.d.	

*Histological tumor grade. (n.d.: as survival in the particular group did not drop below 50%). n.d., not determined.

**Table:** 

*OS*	*all*		grade III*		grade IV*		AA, AOA, AO *IDH1*mut		AA, AOA, AO *IDH1*wt	
*XAF1*	mean	median	mean	median	mean	median	mean	median	mean	median
UM	16.8	12.0	21.9	19.0	15.3	11.0	-	-	16.4	11.0
M	40.2	n.d.	49.8	n.d.	20.6	16.0	54.2	n.d	-	-
p	0.000		0.000		0.196		n.d.		n.d.	

*Histological tumor grade. (n.d.: as survival in the particular group did not drop below 50%). n.d., not determined.

Mean and median survival estimates according to the *XAF1* methylation status for PFS and OS in different subgroups of the patients, collectively analyzed. *P*-values indicate the statistical significance of the differences in both groups (log-rank test).

According to the new WHO classification published in 2016, WHO grade III AA, AO and AOA have to be further classified according to their *IDH1* status [4]. In AA, *IDH1*-wild-type (*IDH1*wt) is an uncommon event and most of these cases share genetic similarities with *IDH1*wt GB [38, 39]. Therefore, the *IDH1* status was determined by IHC using an anti-IDH1 R132H antibody (Dianova) and was additionally validated by pyrosequencing. As expected, strongly increased PFS and OS (*p* < 0.0001) were observed for *IDH1*mut patients (Supplementary Figure 2A). The data indicate that among the 26 grade III tumors, 17 tumors showed the R132H mutation by both, IHC and pyrosequencing. Eight tumors showed an *IDH1*wt status by both techniques. One tumor was diagnosed as *IDH1*wt by IHC but pyrosequencing revealed a rare heterozygous mutation leading to an arginine → glycine (R132G) substitution (Supplementary Figure 3). According to the 2016 WHO guidelines, these eight tumors were defined as a separate class (grade III, *IDH1*wt). As a result, all grade III, *IDH1*mut tumors also showed methylation of *XAF1* (Table 1).

In GB, only two out of eight *XAF1*-M tumors were *IDH1*mut. In addition, these two have been histologically characterized as secondary GB, derived from AO. The *IDH1 and IDH2* status of the six GB samples was verified by pyrosequencing. Within these *IDH*wt GB, *XAF1*M tumors may form a distinct group. Due to the low number of these tumors in our cohort (*N* = 6), no difference, however, was observed for PFS and OS for *XAF1* methylation (Supplementary Figure 2B).

The high association between *IDH1* and *XAF1* status was also observed in recurrent gliomas (Table 3, Supplementary Table 2). Among 16 recurrent gliomas, all six recurrences derived from astrocytomas are *IDH1*mut*/XAF1-*M. From the ten recurrent GB, eight are *IDH1*wt*/XAF1-*UM and two are *IDH1*mut*/XAF1-*M, which points to their origin from a lower grade tumor. Indeed, these *IDH1*mut*/XAF1-*M tumors have derived from AA.

**Table 3:** *XAF1* promoter methylation status and *IDH1* status in *HGG* patients

	N	*XAF1*-M% (N)				N	*XAF1*-M% (N)				N	*XAF1*-M% (N)	
All	80	32.5 (26)			Grade III	26	69.2 (18)		Grade IV		54	14.8 (8)	
*IDH1*wt	60	10.0 (6)			*IDH1*wt	8	0.0 (0)		*IDH1*wt		52	11.5 (6)	
*IDH1*mut	20	100.0 (20)			*IDH1*mut	18	100.0 (18)		*IDH1*mut		2	100.0 (2)	

*Cases (N)*			*XAF1*-M	*XAF1*-UM		*IDH1*mut		*IDH1*wt		*XAF1*-M*IDH1*mut			*XAF1*-M*IDH1*wt	
Grade III* (26)			18	8		18		8		18			0	
Grade IV* (54)			8	46		52		2		2			6	
Grade III (18) *IDH1*mut			18	0		18		0		18			0	
Grade III (8) *IDH1*mut			0	8		0		0		0			8	
Grade IV (2) *IDH1*mut			2	0		2		0		2			0	
Grade IV (58) *IDH1*mut			6	52		0		58		0			6	
Recurrent grade III (6)			6	0		6		0		6			0	
Recurrent Grade IV (10)			2^+^	8		2^+^		8		2^+^			0	

*Histological tumor grading. ^+^derived from OA.

## DISCUSSION

HGG are the most common and aggressive type of primary brain tumors. In our previous studies, we showed that cytotoxicity of the topoisomerase I inhibitor topotecan is strongly affected by the IAPs Survivin and XIAP [17] protecting GB cells from induction of apoptosis. A factor that counteracts these IAPs is the tumor suppressor XAF1. *In vitro* studies showed that XAF1 suppresses tumor cell growth and enhances the cellular response to various apoptotic stimuli [30]. Here we show that XAF1 has an impact on TMZ-induced apoptosis, as XAF1 knockdown in the GB cell line LN229 resulted in a decline of apoptotic frequency. In TMZ-exposed XAF1 knockdown cells we observed a switch in the cell population from the G2- to the G1-phase, which suggested that these cells accumulate in G1 where they presumably get arrested for a while. In this case the G1-arrested cells will not form colonies, which could explain the missing difference in the colony formation of *XAF1* knockdown cells. As already mentioned, this could indicate that for long-term survival differences (as in glioma patients under TMZ therapy), the impact of XAF1 on cell cycle progression might play a predominant role.

XAF1 was previously shown to induce apoptosis independent of XIAP inhibition. Thus, XAF1 enhances p53 expression by antagonizing inhibition of p53 by MDM2 and increases HIPK2-dependent phosphorylation of p53 at Ser46 [40]. Phosphorylation of p53 at Ser46 leads to the transcription of pro-apoptotic genes and the execution of apoptosis [41]. In contrast, Ser15 phosphorylation drives the expression of the cell cycle regulator p21 [42] which is involved in the regulation of the p53-induced growth suppression [43, 44]. Lee *et al*. demonstrated a XAF1-induced down-regulation of p21 *via* stabilization of the p21-targeting E3 ubiquitin ligase ZNF313 [40] switching from cell cycle arrest to the induction of apoptosis. Furthermore, enhanced XAF1 expression was shown to inhibit cell proliferation and induce apoptosis, which was mainly associated with the induction of the G2/M arrest [33]. Cell cycle arrest in G2/M is explained by a direct interaction with CHK1 [34].

**Figure 5:**
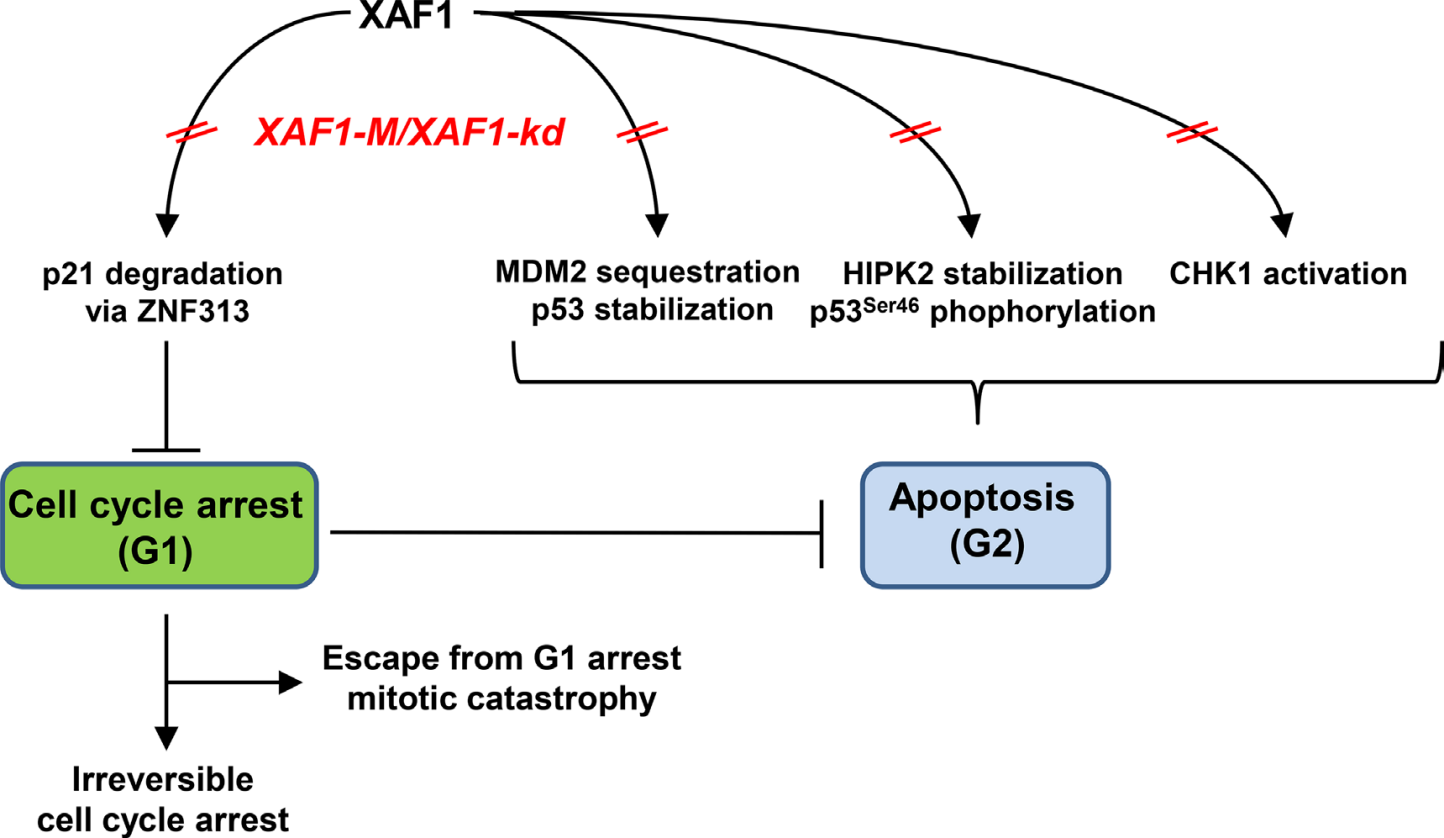
Potential impact of XAF1 on the decision between apoptosis and cell cycle progression upon TMZ treatment

Taking the available data together, we suggest the following model. In TMZ- treated GB cells, XAF1 plays a role in the decision between apoptosis and cell cycle arrest. XAF1 down-regulation attenuates activation of CHK1 and therefore prevents from phosphorylation of p53 at Ser46, thereby abrogating the TMZ-induced G2-arrest and apoptosis. By inducing the G1-arrest resulting from an elevated activation of p21, persistent reduced levels or absence of XAF1 could arrest the DNA synthesis, thereby preventing the replication- and mismatch repair-dependent induction of DSB [15, 16], further reducing the apoptotic frequency (Figure 5). It is conceivable that this scenario has two outcomes: (i) the tumor cells either become irreversibly arrested in the G1/S-phase or (ii) they can progress at late times with newly formed DSB through S and G2 into mitosis, followed by induction of mitotic catastrophe. Both outcomes would result in tumor regression, leading to improved clinical outcome of *XAF1*-M tumors upon TMZ therapy. Since *XAF1*-M correlates with *IDH1* mutation, *XAF1* silencing could contribute to the enhanced survival of patients with *IDH1* mutations. Those cell cycle effects might be overshadowed by the overall G-CIMP in grade III *IDH*mut patients. Therefore, it is essential to analyze the impact of *XAF1* methylation in GB with *IDH1*wt status. The proposed model provides implications that need to be verified by further experiments.

As epigenetic silencing of *XAF1* occurs in different tumor entities [21, 24, 29, 45], we further addressed the question whether the *XAF1* promoter methylation is associated with clinical outcomes in HGG patients. A clinical impact of the XAF1 expression was already shown in epithelial ovarian cancer [22], pancreatic tumors [23], clear-cell renal cell cancer [27], and gastric adenocarcinomas [46]. In all cases, a lower XAF1 expression was associated with poor prognosis. Due to the tumor-suppressing nature of XAF1, we anticipated a similar impact in HGG. Unexpectedly, methylation of the *XAF1* promoter was found to be significantly correlated with an improved OS and PFS in a collective of 80 HGG patients. To analyze the promoter methylation, we established a sensitive and quantitative high-throughput method (MS-HRM). Stratifying according to tumor histology revealed that the survival difference was specific for grade III gliomas (AA, AO and AOA) and suggested *XAF1* methylation to be a prognostic marker for these tumor entities. However, since diffuse gliomas represent a problem in the classification, different genetic markers have been tested, and the results have been integrated into the 2016 WHO classification guideline [4]. Thus, all diffuse gliomas have to be classified according to the *IDH* status. In the case of AA, *IDH1*wt astrocytomas share a high genetic identity with GB [39, 45]. Therefore, it is highly important to separate these subtypes. For detection of *IDH1* mutations, the use of pyrosequencing in addition to conventional IHC detection is demanded for reliable classification. The most common (> 95%) *IDH1* mutation leads to an R132H substitution [47], which causes a gain-of-function in the IDH1 enzyme. However, also other mutations at this position (R132C, R132S, R132G, R132L, R132V, R132P) have been reported [48].

Classifying the grade III tumors according to their *IDH1* (R132H) status by IHC revealed that among these 26 tumors nine showed the *IDH1*wt status. On the contrary, 17 tumors carried a mutation in *IDH1* and were additionally methylated in *XAF1*. Strikingly, among the *IDH1*wt tumors, only one tumor was found to be *XAF1*-M. Importantly, we identified this patient with an AA as a carrier of a rare *IDH1* mutation (R132G), not detected by IHC. The false-negative *IDH1* status of this patient could be re-classified by pyrosequencing according to the *XAF1* methylation. Thus, we were able to show an absolute correlation (18 out of 18) between the *XAF1* methylation and the occurrence of 2-HG-producing *IDH1* mutations, within grade III HGG (4 AO, 7 AOA, 7 AA). *Vice versa*, the eight *IDH1*wt grade III tumors had an unmethylated *XAF1* promoter. However, as discussed earlier, *IDH1*wt tumors with grade III histology show more similarities to the tumor entity of *IDH1*wt GB (grade IV) [4]. This has also been observed in the patients’ survival. While the mean OS of patients harboring *IDH1*wt*/XAF1-*UM tumors was 16.4 months, patients with *IDH1*mut*/XAF1-*M AA/AOA had an OS of 54.2 months (Table 2). The low OS indicates that these *IDH1*wt*/XAF1-*UM tumors might actually represent grade IV GB. According to the 2016 WHO classification, this tumor group should be considered with caution, as it might resemble other tumor entities of higher grade.

In GB, only eight out of 54 tumors were *XAF1*-M. Two out of the eight *XAF1*-M tumors were also *IDH1*mut. Interestingly, tumor histology indicated that these *IDH1*mut*/XAF1-*M tumors are secondary GB as they have been derived from OA of a lower grade. However, six *XAF1*-M GB did not show an *IDH1* mutation at R132. To exclude a 2-HG-producing *IDH2* mutation as well, we sequenced the corresponding position at R172 and could detect the *IDH2*wt sequence in all six samples (Supplementary Figure 6). By the exclusion of the most frequent 2-HG-producing mutations R132H, R132C, R132G, R132S, and R132L in *IDH1* [48], and verification of the *IDH2* R172 wt status [6] by pyrosequencing, we deduced that in GB, *XAF1* methylation can also occur independent of *IDH1* R132 and *IDH2* R172 mutations. However, since not all mutation spots in the *IDH1* and *IDH2* genes producing 2-HG (e.g. *IDH2* R140) have been determined and since 2-HG production was not directly measured, it should be clarified whether *XAF1* methylation can also be caused by these rare mutations. Thus, *XAF1* methylation might provide an additional prognostic and/or predictive value for this tumor entity. However, since the number of *IDH1*wt/*XAF1*-M tumors within the data set was very low, no conclusion concerning the impact of the *XAF1* status on the survival of these patients can be drawn yet.

The co-occurrence of *IDH1* mutations and *XAF1* methylation in grade III tumors (AA, AO, and AOA) indicates that the promoter methylation of *XAF1* might be a consequence of 2-HG produced in *IDH1* mutated cells. This hypothesis is supported by data from Turcan *et al*. [9] analyzing differentially methylated genes in *IDH1*-R132H expressing human astrocytes. Here, a 7.35-fold increased methylation in CpGs belonging to *XAF1* promoter can be found. Furthermore, comparing CIMP-positive *vs*. CIMP-negative tumors, a 2.63-fold repression of mRNA expression and a 3.23-fold enhanced methylation of *XAF1* was observed in the MSKCC cohort of lower grade glioma samples [9]. In a recent study on the methylation profile of the GB samples from the TCGA (The Cancer Genome Atlas) [38], a significant down-regulation of *XAF1* expression and hypermethylation was detected in G-CIMP proneural gliomas. Additional expression data of *XAF1* in G-CIMP-positive GB are, to our best knowledge, not available. Of note is that the G-CIMP phenotype is associated with *IDH1* mutations in gliomas. The coincidence of *XAF1*-M and *IDH1*mut in grade III gliomas explains why this subgroup shows a better survival despite opposite results (for the loss of XAF1) obtained in other tumor types. Therefore, *XAF1* methylation does not represent an independent prognostic marker in this particular tumor entity but provides a surrogate marker of *IDH1* and probably *IDH2* mutations. The MS-HRM-facilitated detection of the *XAF1* methylation, presented in this study, thus could provide a fast and cheap diagnostic tool for assessing the *IDH* status in tumor samples. Clinically, the *IDH1* status is determined *via* pyrosequencing, which is more expensive and requires several working steps following DNA isolation. Different primer sets have to be established for *IDH1* and *IDH2* for each region of interest. Since about 95% of *IDH1* mutations are R132H substitutions, IHC with IDH1R132H mutation-specific antibodies is most commonly used for diagnosis but might miss rare *IDH1* mutations and does not detect *IDH2* mutations. Also, discrepancies between IHC and DNA sequencing have been reported [49, 50]. Here, *XAF1*-targeted MS-HRM could provide a cheap and error-free high-throughput analysis for the detection of 2-HG-producing *IDH* mutations. While we could provide evidence that *XAF1* methylation is strictly (100%) linked to 2-HG-producing mutations in *IDH1* (shown for R132H, R132G) in grade III gliomas, we were not able to identify a less frequent *IDH2*-mutant tumor within the data set. Thus, we can only speculate that 2-HG-producing *IDH2* mutations might have the same impact on *XAF1* methylation. Before considering *XAF1*-targeted MS-HRM as surrogate detection method for *IDH* mutations in gliomas, this association would have to be proven in a larger cohort.

Considering XAF1 as tumor suppressor, the link between *XAF1*-M and an improved OS/PFS might be attributed to the overall G-CIMP effects, which themselves are associated with a better prognosis. However, the exact nature of this phenotype is not understood in detail and does not provide a specific molecular explanation for the observed survival benefits. In either case, the initially anticipated disadvantageous effects of *XAF1* silencing for the survival were disproved. The same was true for the influence of *XAF1* expression/*XAF1* silencing on expression of its interaction partner XIAP. Since XIAP is post-translationally regulated by XAF1, a reduced XIAP level in *XAF1*-UM tumors could have been assumed. Thus we analyzed the protein level of XIAP in representative tumor samples (*XAF1*-M *vs*. *XAF1*-UM). IHC staining showed no differences in the levels of XIAP in either case (Supplementary Figure 5).

In summary, we could show that *XAF1* methylation can occur independently of *IDH1* R132 and *IDH2* R172 mutations. *IDH*2 R140 mutations characterized by a moderate 2-HG production have not been analyzed here. As no significant difference in survival of this small group of patients with *IDH*wt/*XAF1*-M could be observed, when comparing with *IDH1*wt/*XAF1*-UM group, a negative influence of *XAF1*-M on the survival cannot be excluded. Thus, an extended screening of primary GB with frequently occurring *IDH*wt genotype as to the promoter methylation state of *XAF1* is necessary to provide valuable information about *XAF1* methylation as an independent biomarker for this tumor entity.

## MATERIALS AND METHODS

### Ethics statement

Investigation has been conducted in accordance with the ethical standards and according to the Declaration of Helsinki and according to national and international guidelines and has been approved by the authors’ institutional review board.

### Patients and treatment protocols

DNA was isolated from formalin-fixed, paraffin-embedded (FFPE) tumor samples from 80 patients with a first diagnosis of a high-grade glioma and from 16 patients with recurrent HGG, treated at the Department of Neurosurgery of the University Medical Center, Mainz, Germany, between February 2011 and June 2013. Tumors had been assigned histologically to gliomas of WHO grade III and IV by a neuropathologist (C. Sommer). Tumor specimens were obtained by resection, performed before initiation of treatment (first diagnoses) and were immediately formalin-fixed and paraffin-embedded. Tumor material was micro-dissected for further analysis and tumor areas were labeled on the slides. In patients with GB and adequate postoperative clinical condition, a combined radio-chemotherapy with TMZ was performed according to the EORTC regimen [14, 51]. In patients with AA°III or AOA°III a radio-chemotherapy according to NOA-04 protocol or alternatively a combined radio-chemotherapy according to the EORTC regimen was performed [52]. In case of tumor progression, second-line therapy was administered, e.g. dose dense TMZ, CCNU or bevacizumab. All patients provided written informed consent. The study was approved by the institutional ethics committee of the University Medical Center Mainz.

### Cell culture

Malignant glioma cell lines (U373, U138, LN308, U343, A172, U251, MO59J, MO59K, D247, LN229, LN18, GBP61, T98G, U118, LN319) were kindly provided by Prof. Weller (Laboratory of Molecular Neuro-Oncology, University Hospital and University of Zurich, Switzerland) and cultured in DMEM (Gibco) supplemented with 10% fetal calf serum (Gibco) and grown at 37°C, 7% CO_2_.

### Preparation of RNA and qRT-PCR

Total RNA was isolated from cultured cells using the NucleoSpin^®^ RNA extraction kit (Macherey-Nagel). The reverse transcription was performed with the Verso cDNA Synthesis Kit (Thermo Fisher Scientific) using random hexamer primers for cDNA synthesis. Real-time PCR was carried out with UltraMastermix (Promega) on a CFX96 Real-Time PCR Detection System (Bio-Rad). The genes *ENOX2* and *ACTB* were used for normalization of the *XAF1* expression. *ENOX2* and *ACTB* primers were obtained from Primerdesign (UK) and expression was verified to be stable among different glioma cell lines with the best keeper software [53]. *XAF1* primers were specially designed for this analysis and were synthesized by Eurofins-Genomics (forward: 5′-AGCAGGTTGGGTGTACGATG-3′ and reverse: 5′-CCTGGCACTCATTGGCCTTA-3′).

### Preparation of protein extracts and western blot analysis

Whole cell extracts were prepared as described [17]. For protein detection primary antibodies were diluted 1:1000 (XAF1: Pro-Sci 3207 & Santa Cruz sc-374020, Survivin: R&D Systems #AF886, XIAP: Becton Dickinson BD #610716, pCHK1/CHK1: Cell Signaling Technology CST #2341 / CST #2360, HSP90: Santa Cruz sc-13119). Appropriate secondary antibodies (1:2000; Rockland) were used for ECL detection (Pierce) or detection on the Odyssey infrared imaging system (1:10000; IRDye 680LT donkey anti-mouse IgG; Licor/ 1:10000; IRDye 800CW anti-rabbit IgG).

### Knockdown of XAF1

1 × 10^5^ cells were seeded per 35-mm dish. After 24 h, knockdown of XAF1 was performed, using siRNA against *XAF1* with a final concentration of 10 μM (Santa Cruz sc-37511) and Lipofectamine RNAiMAX Transfection Kit (Invitrogen).

### Determination of apoptosis, cell cycle distribution and metabolic competence

For analysis of the sub-G1 (apoptosis), G1, S and G2 fractions cells were harvested and fixed at the indicated time points after TMZ treatment as described and were then analyzed by flow cytometry [17, 54]. For unbiased analysis of the cell cycle distribution ModFit LT 3.3 Software was used for the calculations (Verity Software). In addition, induction of apoptosis and necrosis was determined by annexin V-FITC and propidium iodide (PI) double staining as described [17]. Cell viability, i.e. metabolic competence, was determined as described earlier [55]. 0.5 mg/ml 3-(4,5-dimethylthiazol-2-yl)-2,5-diphenyltetrazolium bromide (MTT) was added to the cells and the cells were incubated for 3 h under normal cell culture conditions. To solubilize the generated formazan crystals, the culture medium was removed and 100 μl DMSO with 0.04 M HCl was added. The absorbance was measured at 570 nm. Metabolic competence was calculated as percentage relative to the untreated control.

### Immunofluorescence staining of FFPE tumor sections

Before staining FFPE tumor sections, specimens were deparaffinized. Sections, mounted on a microscopic slide, were pre-heated at 60°C for 30 min and afterwards incubated first in xylene (3 × 5 min) and then in ethanol series (100/100/96/90/80/70%). Rehydration was carried out by rinsing the sections 2 × in H_2_O plus 1 × in PBS. Specimens were incubated in pre-heated citrate buffer (Target Retrieval Solution, Dako GmbH, Hamburg) in a steamer for 20 min. For additional 20 min, the samples were allowed to cool down at RT. After rinsing 2 × in PBS, the sections were subjected to immunofluorescent staining. After blocking for 3 h with blocking solution (Dako GmbH) in a humidified chamber at RT, samples were incubated with XIAP Ab (1:50; BD #610716) in PBS/2% BSA/0.1% TritonX-100 overnight at 4°C. Incubation with secondary A488-conjugated Ab (1:500; Invitrogen A-11017) in PBS/2% BSA was performed for 2 h at RT. Samples were washed (3 × 10 min with PBS/0.1% Tween–20), rinsed 1 x in PBS, stained with TO-PRO–3 (1:100) for 30 min and preserved with Vectashield Antifade Mounting Medium (Vector Laboratories Inc.).

### Isolation of genomic DNA and bisulfite conversion

DNA from cell lines and FFPE samples was isolated using a phenol-chloroform, isoamyl alcohol (25:25:1) protocol followed by ethanol precipitation as described earlier [56]. The extracted DNA was resolved in DNAse-free water and nucleic acid purity and concentration was determined on a spectrophotometer (NanoDrop 2000c; Thermo Fisher Scientific). Absorbance quotients A_260/280_ of ~ 2.0 and A_260/230_ ratios of 2.0–2.2 were generally accepted as pure and DNA was stored for further use at −20°C. To address differences in CpG DNA-methyation, 500 ng DNA were subjected to bisulfite conversion, using the EZ DNA Methylation^™^ Kit (Zymo Research) according to the manufacturer's protocol.

### Analysis of the XAF1 promoter methylation by MS-HRM analysis

Methylation-sensitive (MS) high-resolution melt (HRM) analysis was performed as described [37]. 20 ng bisulfite converted DNA were amplified in duplicates by qPCR using a CFX96 Real-Time PCR Detection System (Bio-Rad). Primers were designed to amplify a 86 bp fragment containing 3 CpGs from -236 to -196 upstream of the XAF1 transcription start site (forward 5′-GGTTGTTAGTTTTAGGGAGGTAGA-3′; reverse 5′-TAGTAGGGGTTGGTTATGTTGT-3′). Melting data were analyzed and normalized using the Precision Melt Analysis Software (Bio-Rad). For sample interpolation, DNA standards with defined overall methylation value (Supplementary Figure 4B) were analyzed in every assay. The area under the curve (AUC) of the normalized melt curves was used to calculate a linear regression model (Supplementary Figure 4C) for the methylation standards (Prism 6.0c for Mac). % methylation was interpolated from the standard curve.

### Analysis of the XAF1 promoter methylation by pyrosequencing

Pyrosequencing was performed on a PyroMark Q96 ID (Qiagen). Bisulfite converted DNA of selected samples was amplified by PyroMark PCR Kit (Qiagen). 1 μg DNA was used with 0.28 μmol/L forward and 5′-biotinylated reverse primer. The biotinylated antisense strand was extracted with sepharose beads and used as template for sequencing reaction with 0.42 pmol/μL sequencing primer.

### Survival analysis

Kaplan-Meier estimates for the PFS and the OS were calculated upon stratification for the *XAF1* methylation status. The survival differences in both groups were tested for statistical significance by log-rank test (Mantel-Cox test). All statistics were computed using SPSS 23 (IBM) and plotted with Prism (version 6.0c for Mac).

### Statistical analysis

Non-parametric Spearman's rank-order correlation was used to calculate the correlation coefficient (r) for the comparison of IDH1 status and *XAF1* methylation. This correlation was tested for significance with a two-tailed test. Pearson correlation coefficient (r) was computed for comparison of *XAF1* methylation values (%) determined by pyrosequencing vs. MS-HRM (Supplementary Table 1) and for the correlation of *XAF1* mRNA expression vs. gene methylation. This correlation was tested for significance with a two-tailed test (SPSS). SubG1, annexin V, and survival (MTT) data were analyzed for statistically significant differences by two-tailed *t-test*, comparing target siRNA (*XAF1-si*) vs. control siRNA (*con-si*) (Prism 6.0c for Mac).

## SUPPLEMENTARY MATERIALS FIGURES AND TABLES


