# Supplementary material for: Epigenetic silencing of XAF1 in high-grade gliomas is associated with IDH1 status and improved clinical outcome
Source: Oncotarget. 2017 Jan 19;8(9):15071–84. doi: 10.18632/oncotarget.14748 (PMC5362468; doi:10.18632/oncotarget.14748)
Supplement: Supplementary file 1 [file oncotarget-08-15071-s001.pdf]

Epigenetic silencing of *XAF1* in high-grade gliomas is associated with *IDH1* status and improved clinical outcome

Supplementary Materials

Analysis of the XAF1 promoter methylation by high resolution melt (HRM) curve analysis

Methylation standards were generated as and bisulfite conversion was performed using the EZ DNA Methylation kit (ZymoResearch). Bisulfite treated DNA was amplified in duplicates by qPCR on a Bio-Rad CFX-96 C 1000 thermocycler. 20 ng DNA were added to 7.5 µl Precision Melt Supermix (Bio-Rad) and 400 nM primers, adjusted with DNase-free water to a total volume of 15 µl. Primers were designed to bind in the promoter region of XAF1 to amplify an 86 bp fragment including 3 CpG-sites. Forward (5′ GGTTGTTAGTTTTAGGGAGGTAGA 3′) and reverse (5′ TAGTAGGGGTTGGTTATGTTGT 3′) primers did not cover any CpG-site, thus did not preferentially bind to either methylated or unmethylated sequences. PCR protocol was setup as follows: 120 s at 95°C, 50 cycles of 10 s 95°C, 30 s 60.3°C, 15 s 72°C. After 30 s 95°C and 60 s at 60°C post-PCR melt curve was recorded with 0.2° increments ranging from 65–90°C. Melting data were normalized and analyzed using Precision Melt Analysis Software (Bio-Rad) (Supplementary Figure 4A). For sample interpolation DNA standards with defined overall methylation value were amplified in every assay. The area under the curve (AUC) of the normalized melt curves (Supplementary Figure 4B) was used to calculate a linear regression of the standard values (GraphPad Prism version 6.0c for Mac). Methylation percentage of the analyzed promoter region was interpolated from standard curve (Supplementary Figure 4C).

For cell lines, each reaction was performed in technical duplicates, and in technical duplicates for the patient samples. Fully methylated and unmethylated bisulfite converted DNA was mixed to obtain standards with a theoretical methylation level of 0%, 25%, 50%, 75% and 100%. Pyrosequencing revealed empirical methylation levels of 1.6%, 21.0%, 42.0%, 71.0% and 84.1%. Standards were included in duplicates in each assay, as well as a non-template control and a genomic DNA control.

1. Switzeny OJ, Christmann M, Renovanz M, Giese A, Sommer C, Kaina B. MGMT promoter methylation quantified by HRM in comparison to MSP and pyrosequencing for predicting high grade glioma response. Clinical epigenetic. 2016; in press.

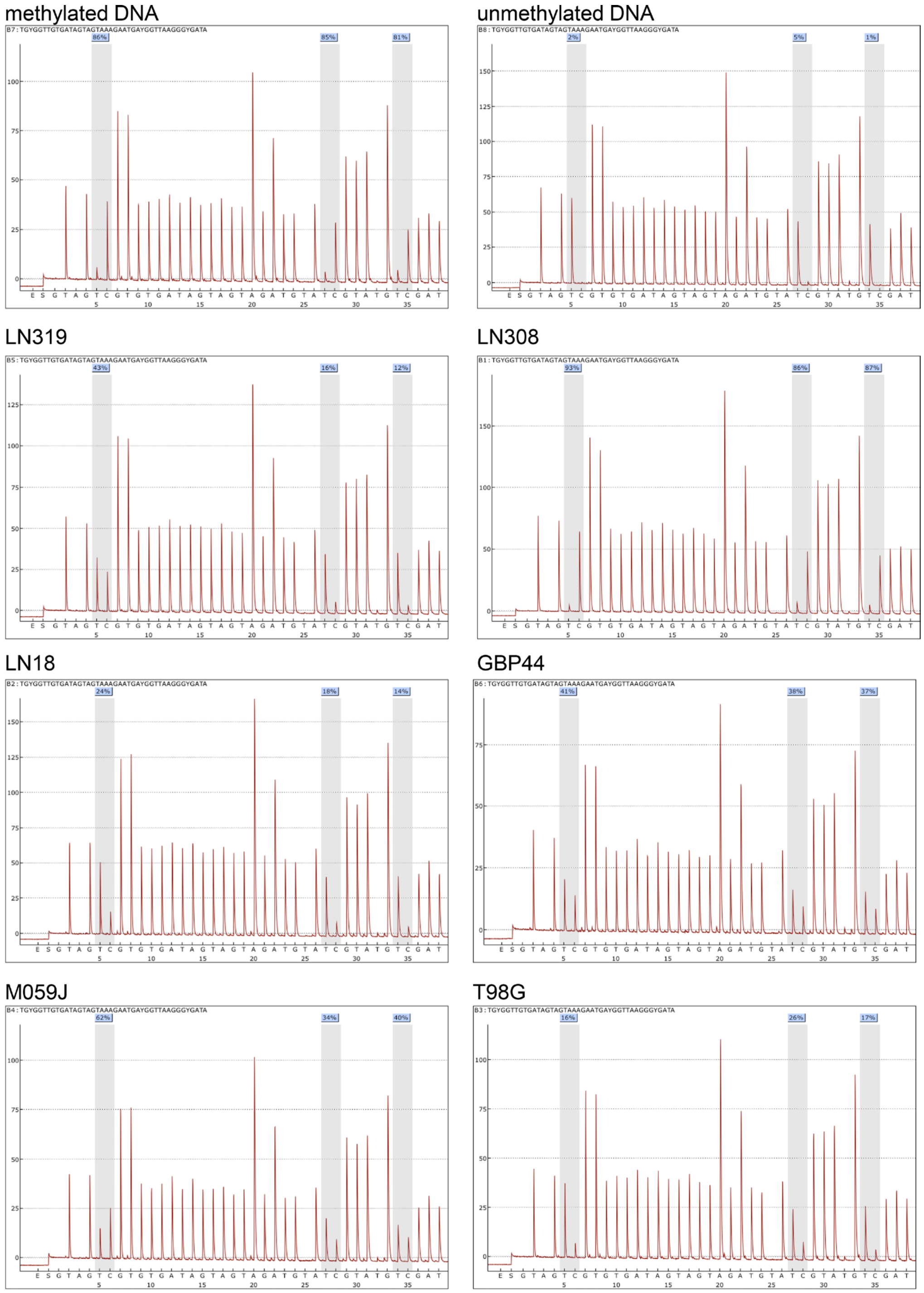

Supplementary Figure 1: CpG methylation within the *XAF1* promoter. Pyrograms of selected cell lines sequenced for C > T transitions (unmethylated CpG sites) in bisulfite converted DNA of glioblastoma cell lines and standard methylated/unmethylated DNA used for MS-HRM analysis sample interpolation. The average methylation percentage was used for verification of the results obtained by MS-HRM.

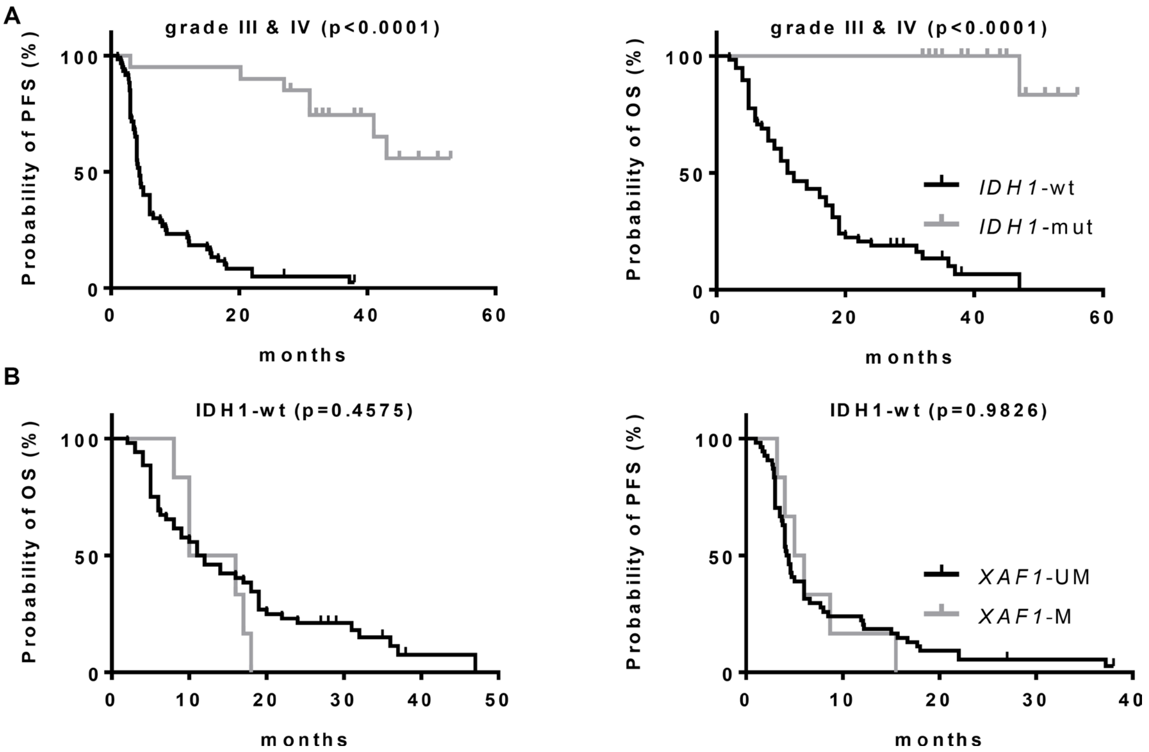

Supplementary Figure 2: Kaplan-Meier survival estimates for HGG glioma patients according to *IDH1* mutation and *XAF1* promoter methylation state. (**A–B**) Kaplan-Meier estimates for PFS and OS of 80 HGG patients. (A) OS and PFS of HGG patients with wt or mutated IDH1, determined by IHC. (B) OS and PFS of IDH1wt HGG patients with unmethylated and methylated *XAF1* status. Significance levels were determined by the log-rank test.

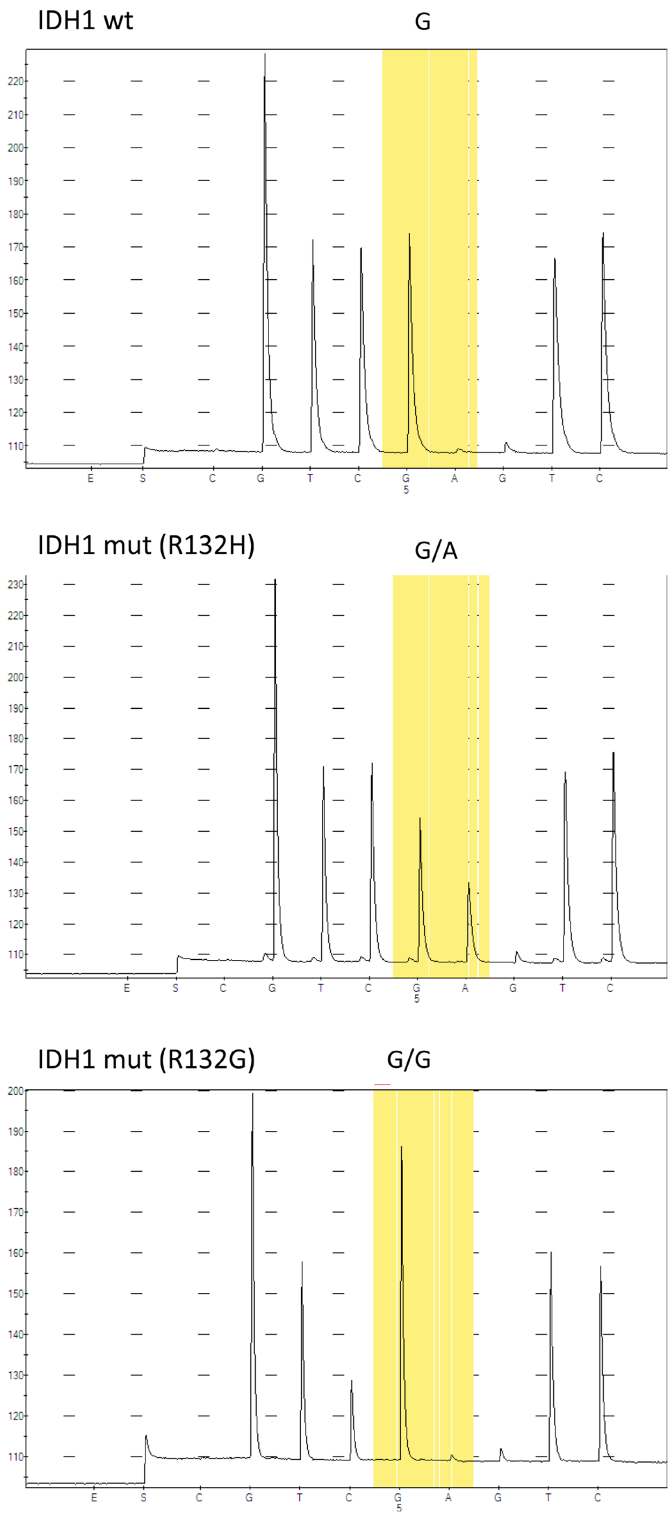

Supplementary Figure 3: Pyrosequencing results for R132-*IDH1*-mutations. Pyrosequencing data are provided for one *IDH1*-wt sample (top), the common heterozygous G395A *IDH1*-R132H mutation (middle) and the rare heterozygous mutation at position 394 (C > G) causing a R132G substitution found in one patient (bottom).

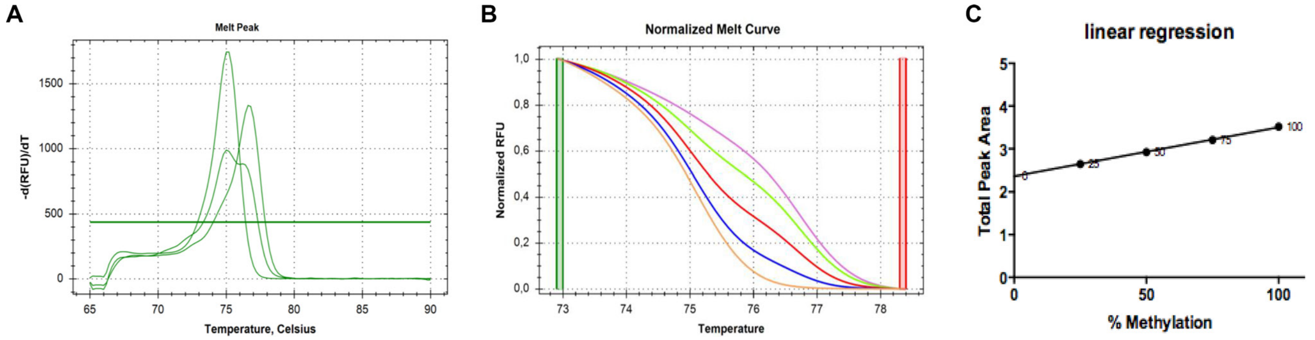

Supplementary Figure 4: MS-HRM analysis. (**A**) Melt curves obtained by qPCR-based MS-HRM analysis for unmethylated (left curve), 1:1 mixture of both standards indicating 50% methylation (middle) and fully methylated DNA-standards (right). (**B**) Normalized melt curves of methylation standards (orange = 0%, blue = 25%, red=50%, green = 75%, pink = 100%) in duplicates. (**C**) Regression model used for XAF1 promoter methylation quantification. Area under the curve (AUC) from the normalized melt curves were used to calculate linear regression ($R^2 > 0.97$) with the corresponding methylation values.

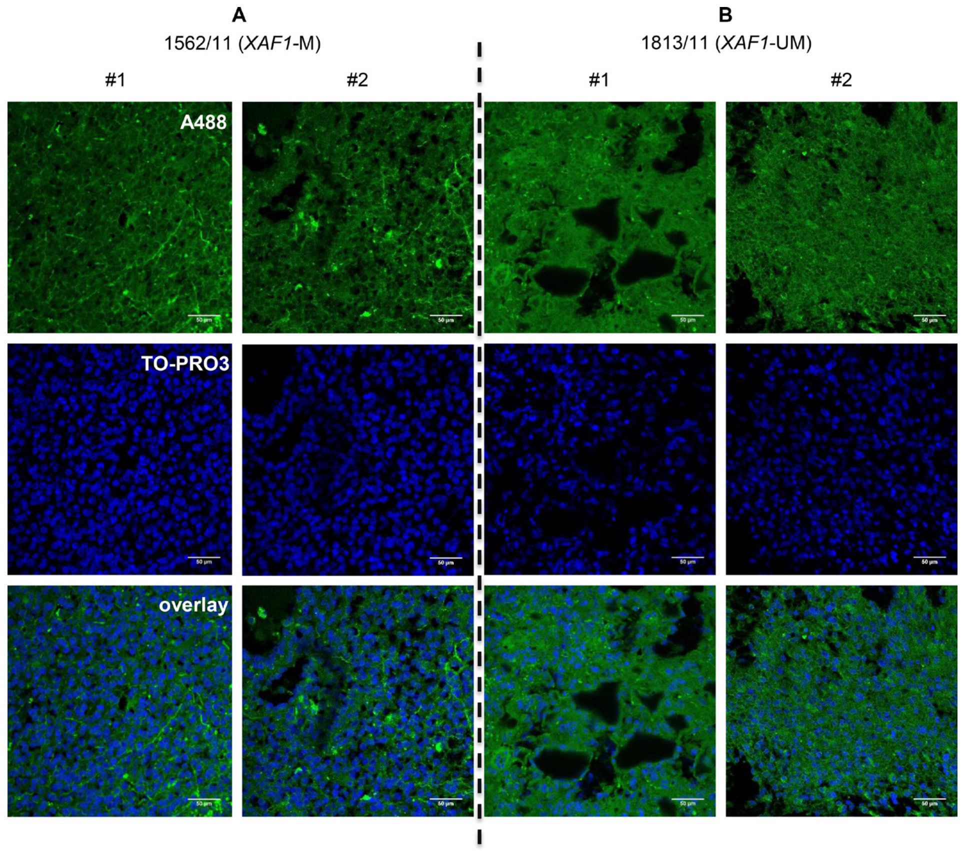

Supplementary Figure 5: XIAP protein level in glioma sections determined by IHC. XIAP protein levels were detected by IHC with a monoclonal Ab against XIAP. Detection was performed by IF with A488-conjugated secondary Ab (green) and TO-PRO3 for nuclear staining (blue). Two different tumor areas (#1; #2) representative for each slide are presented for one *XAF1*-M grade III tumor (**A**) and one *XAF1*-UM grade IV GB (**B**).

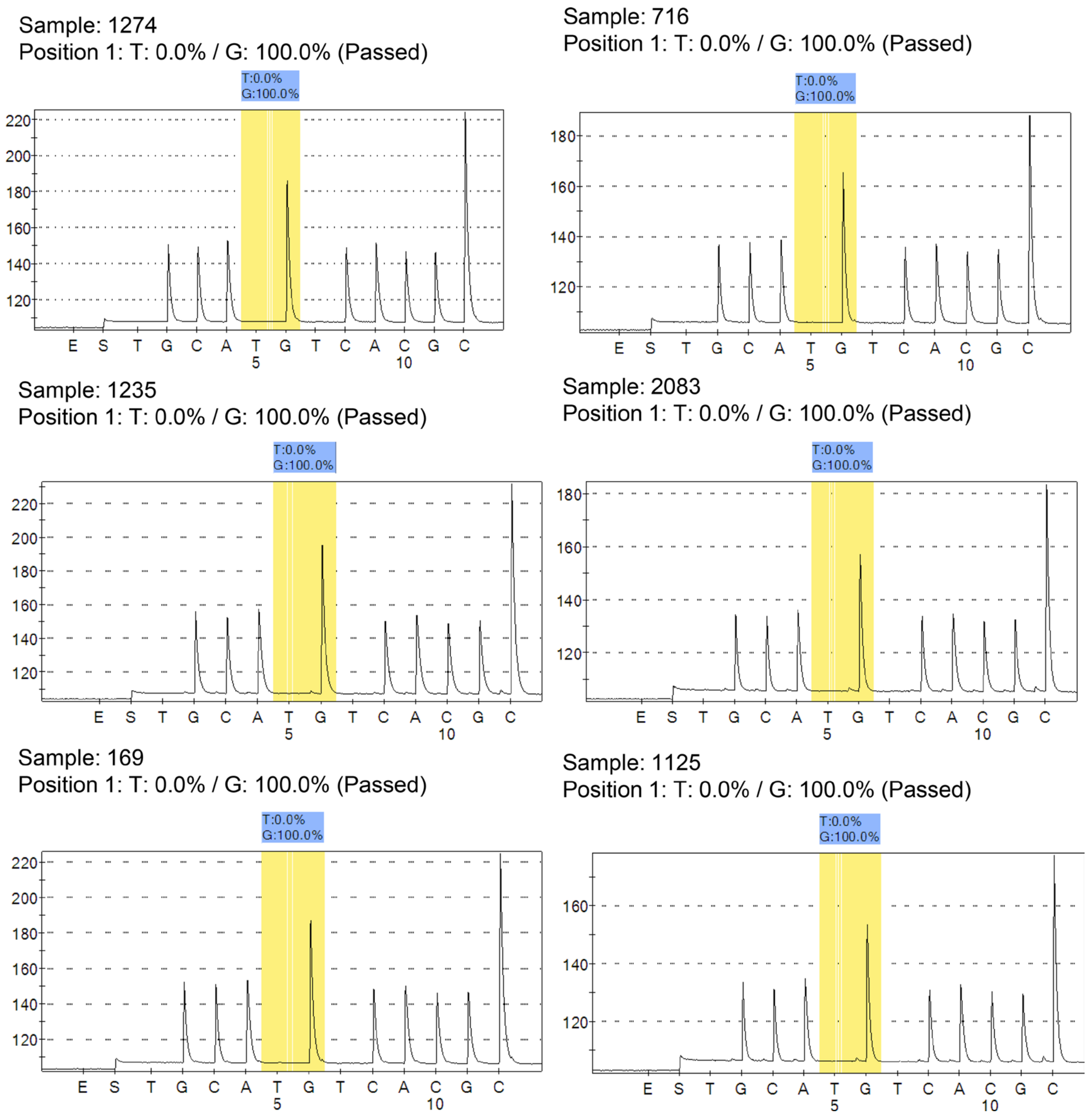

Supplementary Figure 6: *IDH2*-R172 pyrosequencing results for six GB patient's samples with *XAF1*-M and *IDH1*wt status. *IDH2*wt status was verified in a patient's subgroup with *XAF1* methylated tumors and *IDH1*wt status. No SNP was detected at position 172 of the *IDH2* gene.

Supplementary Table 1: Comparison of CpG methylation determined by pyrosequencing (CpG 1–3 and mean) and HRM analysis

	CpG1 (meth %)	CpG2 (meth %)	CpG3 (meth %)	mean (meth %)	HRM (meth %)
**LN308**	92.7	85.6	87.2	88.5	90.3
**LN18**	23.9	18.5	13.9	18.8	2.5
**T98G**	16.4	26.3	16.7	19.8	13.1
**M059J**	62.5	33.7	40.0	45.4	48.9
**LN319**	42.5	16.3	12.1	23.7	8.3
**GBP44**	40.8	38.2	37.3	38.8	48.9

Both methylation values show a strong positive correlation ($r = 0.965$; $p = 0.0018$).

Supplementary Table 2: *IDH1*/*XAF*1 status in recurrences

Patient	Histological grading	*XAF1* status	*IDH*1 status
**1**	AOA°III	*XAF1*-M	*IDH1*mut
**2**	AOA°III	*XAF1*-M	*IDH1*mut
**3**	AOA°III	*XAF1*-M	*IDH1*mut*
**4**	AA°III	*XAF1*-M	*IDH1*mut*
**5**	AA°III	*XAF1*-M	*IDH1*mut
**6**	OA°III	*XAF1*-M	*IDH1*mut
**7**	GBM°IV	*XAF1*-UM	*IDH1*wt
**8**	GBM°IV	*XAF1*-UM	*IDH1*wt
**9**	GBM°IV	*XAF1*-UM	*IDH1*wt
**10**	GBM°IV	*XAF1*-UM	*IDH1*wt
**11**	GBM°IV	*XAF1*-UM	*IDH1*wt
**12**	GBM°IV	*XAF1*-UM	*IDH1*wt
**13**	GBM°IV	*XAF1*-UM	*IDH1*wt
**14**	GBM°IV	*XAF1*-UM	*IDH1*wt
**15**	GBM°IV	*XAF1*-M	*IDH1*mut**
**16**	GBM°IV	*XAF1*-M	*IDH1*mut**

*derived from astrocytoma grade II; **derived from astrocytoma grade III.
